# Novel suspension retroviral packaging cells generated by transposition using transposase encoding mRNA advance vector yields and enable production in bioreactors

**DOI:** 10.3389/fbioe.2023.1076524

**Published:** 2023-04-04

**Authors:** Yasemin van Heuvel, Stefanie Schatz, Marc Hein, Tanya Dogra, Daniel Kazenmaier, Natalie Tschorn, Yvonne Genzel, Jörn Stitz

**Affiliations:** ^1^ Research Group Medical Biotechnology and Bioengineering, Faculty of Applied Natural Sciences, University of Applied Sciences Cologne, Campus Leverkusen, Cologne, Germany; ^2^ Institute of Technical Chemistry, Gottfried Wilhelm Leibniz University Hannover, Hanover, Germany; ^3^ Chair of Bioprocess Engineering, Otto-Von-Guericke-University Magdeburg, Magdeburg, Germany; ^4^ Max Planck Institute for Dynamics of Complex Technical Systems, Bioprocess Engineering, Magdeburg, Germany; ^5^ Faculty of Biotechnology, University of Applied Sciences Mannheim, Mannheim, Germany

**Keywords:** sleeping beauty transposon, mRNA transfection, suspension cell, retroviral vector, murine leukemia virus (MLV), stirred-tank bioreactor, gene therapy, mRNA transfection

## Abstract

To date, the establishment of high-titer stable viral packaging cells (VPCs) at large scale for gene therapeutic applications is very time- and cost-intensive. Here we report the establishment of three human suspension 293-F-derived ecotropic MLV-based VPCs. The classic stable transfection of an EGFP-expressing transfer vector resulted in a polyclonal VPC pool that facilitated cultivation in shake flasks of 100 mL volumes and yielded high functional titers of more than 1 × 10^6^ transducing units/mL (TU/mL). When the transfer vector was flanked by transposon terminal inverted repeats (TIRs) and upon co-transfection of a plasmid encoding for the transposase, productivities could be slightly elevated to more than 3 × 10^6^ TU/mL. In contrast and using mRNA encoding for the transposase, as a proof of concept, productivities were drastically improved by more than ten-fold exceeding 5 × 10^7^ TU/mL. In addition, these VPC pools were generated within only 3 weeks. The production volume was successfully scaled up to 500 mL employing a stirred-tank bioreactor (STR). We anticipate that the stable transposition of transfer vectors employing transposase transcripts will be of utility for the future establishment of high-yield VPCs producing pseudotype vector particles with a broader host tropism on a large scale.

## Introduction

Retroviral and lentiviral vectors represent more than 25% of all viral vectors used in somatic gene therapy today (Ginn et al., 2018). Retroviral vectors mediate efficient stable gene transfer into a variety of cell types including early progenitor and hematopoietic stem cells. This qualifies these vectors to be the favorite choice for the treatment of inherited monogenic diseases. The majority of current clinical trials aim at the treatment of adenosine deaminase-deficient severe combined immunodeficiency (ADA-SCID; (Blaese et al., 1995; Aiuti et al., 2002; Gaspar and Thrasher, 2005); X-linked severe immunodeficiency (SCID-X1 (Cavazzana-Calvo et al., 2000; Howe et al., 2008; Hacein-Bey-Abina et al., 2014; Cavazzana et al., 2016); or Wiskott-Aldrich syndrome (WAS (Boztug et al., 2010; Braun et al., 2014; Hacein-Bey Abina et al., 2015; Ferrua et al., 2019)).

Gamma-retroviral vectors based on murine leukemia virus (MLV) can be produced continuously employing stable viral packaging cells (VPCs) expressing the viral structural genes *gag/pol* (packaging construct), *env* (envelope construct) and a transfer vector harboring the gene of interest *in trans* (Maetzig et al., 2011). Most commonly, a transfer vector-free clonal VPC is first generated by screening numerous cell clones for particle production efficiencies (Miller, 1990; Cosset et al., 1995; Morita et al., 2000; Wang et al., 2015). In a second step, the transfer vector of choice is stably transfected followed again by a time-intensive screening of cell clones yielding high-titer vector preparations. To date, mostly adherent VPCs derived from human cell lines are used for clinical grade vector productions hampering the scale-up for preclinical and clinical trials (Coroadinha et al., 2010; Park et al., 2018). In contrast, VPCs that grow in suspension at higher densities as well as in serum-free media allow for viral vector productions in large bioreactors. In pioneering studies, Ghani and colleagues (Ghani et al., 2006; Ghani et al., 2007) established a retroviral packaging cell line derived from a human suspension 293SF cell producing retroviral titers of up to 4 × 10^7^ transducing units per mL (TU/mL) comparable to yields obtained with adherent VPCs. However, a very time-intensive screening needed to be conducted to identify a high-yield transfer vector-positive VPC clone.

We previously reported on the generation of a stable polyclonal VPC using *Sleeping Beauty* (SB)- derived transposon vectors encompassing MLV-derived retroviral vector components, namely, an enhanced green fluorescent protein (EGFP) encoding transfer vector (pSB-LEGFP-N1), a packaging (pSB-Gag/Pol) and an ecotropic envelope construct (pSB-Env) were co-transfected with a transposase expression vector (pSB100X). Within 3 weeks, stable human adherent, as well as suspension VPCs were generated. These VPCs produced MLV-based vectors at high titers efficiently transducing murine-/and hamster cell lines, murine hematopoietic stem and early progenitor cells (HSPCs) as well as cell lines from different donor species recombinant expressing the murine cationic amino acid transporter (mCAT; (Berg et al., 2019; van Heuvel et al., 2021). Here, we describe the establishment of a polyclonal 293-F derived human suspension cell called MuPACK.e in only 3 weeks employing SB- and MLV-based packaging components.

Moreover, we examined whether the time-intensive screening for high-titer cell clones upon introduction of a transfer vector can be omitted. Therefore, we compared three different approaches: MuPACK.e cells were i) stably transfected with the transfer vector plasmid pLEGFP-N1 harboring the reporter genes *egfp* and *neomycin resistance* (*neoR*) - the most commonly used approach. ii) The transfer vector plasmid now encompassing the TIRs of SB flanking the transfer vector cassette (pSB-LEGFP-N1) was co-transfected with the transposase-expression plasmid pSB100X construct. iii) To increase biosafety and to exclude the genomic integration of pSB100X, and thus the potential sustained expression of the transposase possibly resulting in the re-mobilization of vector components, we co-transfected pSB-LEGFP-N1 together with *in vitro* transcribed mRNA encoding the highly active SB100X (Bire et al., 2013; Kebriaei et al., 2017; Tschorn et al., 2022). Subsequently, all 3 cell pools were selected for high transfer vector expression using escalating concentrations of neomycin (G418). Functional and physical vector titers were assessed conducting transduction experiments and vector particle quantification using capsid-specific ELISA and a quantitative reverse transcription PCR (RT qPCR). The most productive VPC established, using transposase transcripts, was further characterized employing enhanced cell densities and larger scale production in an automated stirred-tank bioreactor (STR).

## Methods and materials

### Cells

Embryonic human kidney suspension FreeStyle™ 293-F cells (Thermo Fisher Scientific, United States) were grown in FreeStyle™ 293 expression medium (Gibco, United States) or Dynamis™ supplemented with 8 mM L-glutamine (for STR, Gibco, United States) or GlutaMAX™ (for shake flasks, Gibco, United States). Cells were cultured at 37 °C, 8% CO_2,_ and at 137 rpm in shake flasks (Thermo Fisher Scientific, United States) using a Minitron shaker incubator (INFORS HT, Switzerland) with an orbit of 5 cm. The adherent NIH/3T3 murine fibroblast target cells (ATCC CRL-1658) were maintained in Dulbecco’s modified Eagle medium high glucose, pyruvate (DMEM; Gibco, Germany), supplemented with 10% fetal bovine serum (FBS; Gibco, United States) at 37°C in a humidified atmosphere at 5% CO_2_. Cell number and viability was accessed using a cell counter (anvajo GmbH, Germany).

### Plasmids

The MLV-based retroviral transfer vector pLEGFP-N1 (Clontech, United States) harbors the *enhanced green fluorescent protein* (*EGFP*) and the *neomycin resistance* (*neoR*) genes. The generation of the transfer vector, the packaging construct, the envelope construct in transposon vector backbones and the transposase construct was described previously (Berg et al., 2019).

The *SB100X* gene was amplified from pCMV-SB100X (Berg et al., 2019) and inserted into pIVTRup (a gift from Ángel Raya (Addgene plasmid #101362; https://n2t.net/addgene:101362; RRID:Addgene_101362). This plasmid served as a template for PCR amplification using primers containing the T7 promoter and polyT tail sequences respectively. The resulting amplicons were subjected to *in vitro* transcription (IVT) of SB100X-mRNA using HiScribe™ T7 ARCA mRNA Kit (NEB, USA) following the manufacturer’s instructions, respectively. After DNase treatment, the mRNA was purified using the Monarch^®^ RNA Cleanup Kit (NEB, United States). RNA purity was confirmed using a Tecan Infinite^®^ and stored in aliquots at −80°C. The final mRNA encompassed the 5′-Cap, the 5′-UTR, the coding sequence of the transposase, the 3′-UTR and the polyA tail (Tschorn et al., 2022).

### Establishment of stable ecotropic MLV-based vector packaging cells MuPACK.e

Packaging cells were generated by co-transfection of 3 × 10^7^ 293-F cells in 20 mL shake flask cultures with 35.6 µg of pSB-gag/pol, 11.9 µg of pSB-env and 2.5 µg of the transposase construct using polyethylenimine transfection reagent (PEI:DNA mass ratio of 3:1; linear PEI, 1 mg/mL, MW 40,000; Polysciences Inc., United States). 9 mL fresh medium was added 3 hours later and a complete medium exchange was performed on the following day. Two days post-transfection, cells were subjected to 4 μg/mL puromycin and 50 μg/mL hygromycin (both InvivoGen, France). Every passage, the concentration of both antibiotics was escalated to a final concentration of 10 μg/mL puromycin and 200 μg/mL hygromycin resulting in a stable VPC bulk population called MuPACK.e within 21 days.

The subsequent transfections with the respective transfer vectors were performed as described in detail (Bauler et al., 2020). For 30 million cells a total amount of 16.5 μg of pDNA/mRNA was co-transfected. A mass ratio of 1:10 (transposase to transfer vector) for the plasmid-based transposase construct and a mass ratio of 1 to 1 for the mRNA encoding the transposase was used (PEI:DNA mass ratio of 2:1 and PEI:mRNA mass ratio of 4:1; linear PEI, 1 mg/mL, MW 40,000; Polysciences Inc., United States). The mRNA-PEI mixes in this case were always prepared in a separate tube and PEI was diluted directly into the mRNA-medium mixture. VPCs stably expressing a transfer vector were subjected to G418 mediated selection pressure 4 days post-transfection at increasing concentrations of G418 ranging from 50 μg/mL to a final concentration of 200 μg/mL. Two weeks post antibiotic selection with G418, all three antibiotics were added at final concentrations of 10 μg/mL puromycin and 200 μg/mL hygromycin and G418. After 3 weeks, for transposition-based transfection and 2 month for classical plasmid-based transfection, and rigorous selection, cells were expanded and cryo-stocks were made and rigorous selection, cells were expanded and cryo-stocks were prepared.

### MLV vector productions in shake flasks and STR

Stable VPCs were seeded at a viable cell density of 2 × 10^6^ cells/mL in 500 mL shake flasks in 100 mL antibiotic-free FreeStyle™ medium or Dynamis™ supplemented with 8 mM GlutaMAX™. After 24 h of production, retroviral vectors were harvested by centrifugation at 100 *g* for 3 min at RT and made cell-free using a PVDF syringe filter with a 0.45 µm pore size (Carl Roth, Germany). Retroviral vector preparations were frozen at −80°C in 1.8 mL aliquots. For high-density VPC cultivations, the stable VPC was seeded at 4 × 10^6^ cells/mL in 250 mL shake flasks in 50 mL Dynamis™ medium supplemented with 8 mM GlutaMAX™ and MLV-based vectors were harvested after 48, 72 and 96 h.

For larger-scale vector production, cultivation in an STR with a 500 mL working volume (DASGIP^®^ Parallel Bioreactor System, Eppendorf AG, Cat. 76DG04CCBB) was performed. The STR was equipped with one inclined blade impeller (three blades, 30°angle, 50 mm diameter) and a macro-sparger. Production parameters are shown in Table 1 Prior to inoculation, the stable VPC MuPACK.e.SB-LEGFP.N1_mRNA_ was thawed and expanded in Dynamis™ medium supplemented with highest concentrations of antibiotics in shake flasks for 2 weeks. When cultures showed viabilities >90% and a density of 2 × 10^6^ cells/mL, cells were centrifuged (300 g, 5 min, RT) and the complete medium was replaced with fresh antibiotic-free Dynamis™ medium. The STR was inoculated with 0.8 × 10^6^ cells/mL and ran at 37°C, pO_2_ ≥ 40%, controlled pH 7.0 (deadband ±0.3), and 150 rpm for 10 days. During cultivation thirteen independent viral vector harvests (5 mL) were taken from the cultivation vessel, made cell-free by centrifugation (3,000 g, 10 min at 4°C) and stored at −80°C. The STR was operated in batch mode.

**TABLE 1:** Operating bioreactor production parameters.

Parameter	Bioreactor DASGIP^®^ eppendorf
Basal medium	Dynamis™ + 8 mM L-glutamine
Initial working volume	500 mL
Agitation	150 rpm
Agitation direction	Downflow
Air flowrate	3 L/h
Initial dissolved oxygen (DO)	89.7%
Temperature	37 °C
pH	7.09 no active control post inoculation
Initial VCD	0.84 × 10^6^ cells/mL

### Viral vector titration and flow cytometry

To assess the viral vector titers produced by the established VPCs, 1.0 × 10^5^ adherent target NIH/3T3 murine fibroblasts were seeded in 2 mL per well in six-well dishes 1 day prior to transduction (Nunc, Wiesbaden, Germany). Dilutions of 1:1,000 and 1:10,000 and of retroviral vector samples produced in shake flasks in total volumes of 1 mL were added to target cells. The following day, 1 mL of fresh cultivation medium was added to transduced cells. Three days post-transduction, the percentage of EGFP-positive cells was analyzed using flow cytometry (S3e, Bio Rad, United States; FlowJo BD Biosciences, United States) and used to detect gene transduction efficiencies. Vector titers described as transducing units per mL (TU/mL) were calculated as follows: titer = (F%/(100 × V_mL_)) × S × D wherein F% is the percentage of GFP-positive transduced cells, S represents the number of seeded target cells on the day of transduction, D the dilution factor and V_mL_ the volume of viral vector in mL (Fehse et al., 2004).

For the viral vector titration using vectors produced in STR, adherent target cells were seeded in 48-well dishes at 1 × 10^4^ cells/well in 0.5 mL 1 day prior to transduction. The medium was removed and vector containing supernatant samples in different dilutions of 1:10, 1:100 and 1:1,000, respectively, in a total volume of 0.25 mL were added to the target cells. Three days post-transduction, cells were analyzed employing flow cytometry to determine the percentage of EGFP-positive cells. Vector titers were calculated as described previously employing supernatant dilutions resulting in gene transfer efficiencies between 1.0% and 10.0% EGFP-positive cells (Salmon and Trono, 2007).

To detect the fluorescence intensities of the three VPCs or transduced target cells expressing the EGFP expressing transfer vector, 1 × 10^6^ cells were centrifuged at 100 *g* for 5 min and the cell pellet was diluted in 1 mL flow cytometry buffer (phosphate-buffered saline (PBS), pH 7.2, 0.5% bovine serum albumin (BSA) and 2 mM EDTA). Prior to flow cytometry, viability was determined using an anvajo cell counter (anvajo GmbH, Germany). A total of 10,000 gated single cells were subsequently analyzed for EGFP expression.

### Quantification of retroviral vectors using an anti MLV p30 immunoassay

To assess the efficiency of viral vector production, total particle concentration (i.e., physical titer) were quantified using a colorimetric MuLV core p30 antigen ELISA kit (Cell Biolabs, Inc. (Cat. VPK-156, United States)). From each cell-free retroviral particle harvest, one sample was used to detect the total p30 concentration. Samples were thawed from −80°C and diluted 1:10,000 in expression medium and assayed in a 96-well plates in duplicate.

### Quantification of transfer vector transcripts in vector particles using RT-qPCR

To quantify the viral vector transfer vector RNA (i.e., physical titer), a real-time reverse transcription quantitative PCR (RT-qPCR) was performed. Cell-free and viral vector-containing cell culture supernatant was used for the extraction and purification of vector RNA according to the manufacturer’s instructions (NucleoSpin^®^ RNA virus kit; Macherey-Nagel, Germany).

A two-step hot start RT-qPCR with sequence-unrelated tagged primers was used to specifically quantify viral vector EGFP mRNA copies (Kawakami et al., 2011). Briefly, an external calibration curve was generated for the EGFP-encoding sequence by amplifying from the pLEGFP-N1 transfer vector template plasmid using the primers: T7-gag/EGFP for 5′- TAA​TAC​GAC​TCA​CTA​TAG​GGA​TGG​TGA​GCA​AGG​GC -3′ and T7-gag/EGFP rev 5′- GCT​AGC​TTC​AGC​TAG​GCA​TCT​TAC​TTG​TAC​AGC​TCG​TCC -3’. 300 ng of the amplicons were *in vitro* transcribed to RNA for 2 h at 37°C using TranscriptAid T7 High Yield Transcription Kit (ThermoFisher Scientific, United States). Transcribed RNA standards were treated with 10 vol% DNase (30 min, 37°C) followed by 10 vol% EDTA treatment (15 min, 65°C) and purified using an RNA isolation kit (Macherey Nagel, Germany).

Subsequently, a hot start reverse transcription PCR was performed. Here, 1 µL of each EGFP mRNA sample and of each generated RNA standard (ranging from 5.0E-07 ng to 5.0E+00 ng), 0.5 µL of dNTPs, 6.5 µL of nuclease-free water and 0.5 µL MLV EGFP tagged RT primer (rev 5′- GCT​AGC​TTC​AGC​TAG​GCA​TCT​TAC​TTG​TAC​AGC​TCG​TCC​A -3)’ was first incubated at 65°C for 5 min and then at 55 °C for 5 min. For cDNA synthesis, 2 µL of 5X RT buffer (Thermo Fisher Scientific, USA), 1.25 µL of nuclease-free water, and 0.25 µL of Maxima H minus reverse transcriptase (Thermo Fisher Scientific, United States) were added and incubated (30 min, 60°C), before the reaction was terminated (5 min, 85°C). The generated cDNA was diluted to 100 µL.

To perform the qPCR, 4 µL diluted cDNA, 5 µL of 2X QuantiNova SYBR green PCR mix (QIAgen, Germany), and 0.5 µL each of 1 μM primers EGFP qPCR for 5′- CTCGCCGACCACTACC -3′ and EGFP tagged qPCR rev 5′- GCT​AGC​TTC​AGC​TAG​GCA​TC-3′ were mixed. For the real-time quantification, samples were subjected to initial denaturation (5 min, 95°C), before 40 amplification cycles (10 s, 95°C; 20 s, 62°C) were carried out. The melt curve analysis was between 65°C and 90°C. For absolute quantification, a regression curve analysis was formulated by plotting the CT values of ten-fold diluted RNA standards against the log10 number of the RNA molecules (Frensing et al., 2014).

### Detection of replication-competent retroviruses (RCRs): GFP marker rescue assay and reverse transcriptase (RT) assay

To ensure that detected gene transfer efficiencies were purely a result of vector-mediated transduction and not caused by the unintended generation of RCRs originating from the recombination of complementary vector components, GFP marker rescue assays were performed in triplicate as previously described in detail (Cosset et al., 1995; Berg et al., 2019; van Heuvel et al., 2021). In addition, NIH/3T3 target cells were exposed to vector preparations, expanded and supernatant of transduced cells was collected after five and 12 days. Subsequently, samples were examined using a reverse transcriptase (RT) assay with a detection sensitivity of 10 pg RT per 40 μL sample (Colorimetric reverse transcriptase assay, Roche, Switzerland) following the manufacturer’s instructions. Supernatants of stable VPCs and NIH/3T3 cells exposed to the supernatant of naïve 293-F cells served as positive controls and negative controls, respectively (data not shown).

### Statistics

An unpaired Student’s t-test was used to calculate *p* values. *p* values of less than 0.05 were considered statistically significant (*(*p* ≤ 0.05), **(*p* ≤ 0.01), ***(*p* ≤ 0.001), ****(*p* ≤ 0.0001)). Graphs and statistics were calculated using GraphPad Prism 7 for Windows 10 software (GraphPad Software, Inc. United States).

## Results

### Generation of stable MuPACK.e based VPCs

**FIGURE 1:**
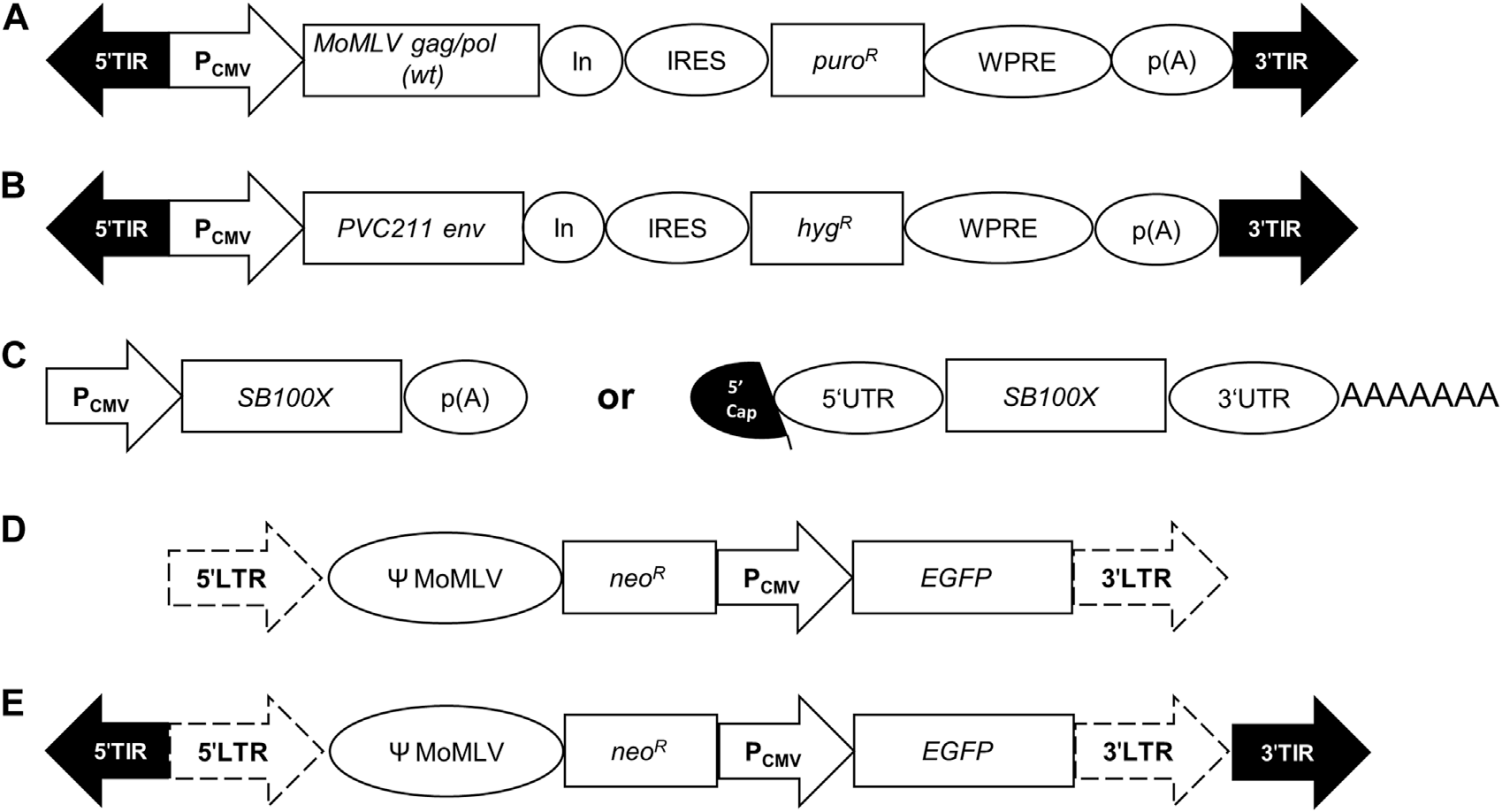
Genetic organization of expression cassettes. **(A)** In the packaging construct pSB-Gag/Pol, a CMV promoter/enhancer element (P_CMV_) drives the expression of the wildtype (wt) Moloney MLV (MoMLV) genes *gag/pol* followed by a synthetic intron (In), an internal ribosome entry site (IRES), a puromycin-resistance gene (*puro* ^*R*^), the Woodchuck hepatitis virus tripartite posttranscriptional regulatory element (WPRE) and the polyadenylation signal (p(A)) of the bovine growth hormone gene. **(B)** The envelope construct pSB-Env encompasses the human codon-optimized ecotropic envelope gene *env* derived from the Friend MLV molecular clone PVC-211 and the hygromycin-resistance gene (*hyg* ^*R*^). (**(C)**, left) The transposase construct pSB100X harbors the human codon-optimized gene of the hyper-active *Sleeping Beauty* transposase SB100X. (**(C)**, right) The transcript mRNA-SB100X encompasses the 5′Cap, the 5′UTR, the coding sequence of the transposase *SB100X*, the 3′UTR and a polyA tail (AAAAAAA). **(D)** The transfer vector pLEGFP.N1 encompasses the 5′- and 3′-long terminal repeats (LTRs; dotted arrows) of murine leukemia virus (MLV) flanking the packaging signal Ψ of MoMLV, a neomycin-resistance gene (*neo* ^*R*^) and a P_CMV_ driven *EGFP* expression. **(E)** The transfer vector pSB-LEGFP.N1 contains the same genetic elements as pLEGFP.N1 but with the flanking 5′- and 3′-terminal inverted repeats (TIRs; black arrows) of *Sleeping Beauty*.

The stable polyclonal suspension VPC MuPACK.e based on ecotropic MLV was established as described using the expression cassettes illustrated in Figure 1, namely, the packaging and envelope construct pSB-Gag/Pol and pSB-Env together with the transposase encoding plasmid pSB100X followed by selection using puromycin and hygromycin. Upon transfection with the constructs illustrated in Table 2 namely, i) only with pLEGFP-N1, ii) with pSB-LEGFP-N1 and the transposase construct and iii) with pSB-LEGFP-N1 and the mRNA of SB100X, stable cell pools were established in the presence of escalating concentrations of G418. Cell-free supernatants of the resultant VPCs MuPACK.e.LEGFP-N1, MuPACK.e.SB-LEGFP-N1 and MuPACK.e.SB-LEGFP-N1_mRNA_ were harvested and frozen at −80°C. Thawed harvests were subjected to three independent titration experiments conducted in triplicate using murine NIH/3T3 target cells. Transduced cells were analyzed 3 days later for EGFP expression.

**TABLE 2:** Overview of plasmids and mRNA employed to generate polyclonal viral packaging cell lines.

VPC:Plasmids, mRNA	MuPACK.e	MuPACK.e. LEGFP-N1	MuPACK.e. SB-LEGFP-N1	MuPACK.e. SB-LEGFP-N1_mRNA_
pSB-Gag/Pol	X	X	X	X
pSB-Env	X	X	X	X
pSB100X	X	X	X	X
mRNA-SB100X	—	—	—	X
pLEGFPN.1	—	X	—	—
pSB-LEGFP.N1	—	—	X	X

### Functional titers of generated MLV-based vectors

As depicted in Table 3 MuPACK.e.LEGFP-N1 generated mean vector titers ranging from 9.63 × 10^5^ to 2.16 × 10^6^ TU/mL. Viabilities of the VPC at the time of vector harvests varied between 76% and 85%. With viabilities of always >90%, MuPACK.e.SB-LEGFP-N1 revealed slightly higher titers of 2.17 × 10^6^ to 3.21 × 10^6^ TU/mL. MuPACK.e.SB-LEGFP-N1 showed stable productivity over a period of 2 months in the presence as well as absence of selection pressure (data not shown). The VPC MuPACK.e.SB-LEGFP-N1_mRNA_ showed the by far highest productivity when cells were cultured in FreeStyle™ medium (harvests 1). Titers of 5.12 × 10^7^ TU/mL were detected at VPC viabilities of 80%, respectively.

**TABLE 3:** Functional titers in TU/mL and physical titers in ng/mL (ELISA) of vector particles harvested from the stable VPCs. Frozen-thawed vector preparations harvested from volumes of 100 mL VPC cultures at viable cell densities (VCD) of 4 × 10^6^ cells/mL in FreeStyle™ medium in 500 mL shake flasks were titrated in triplicate in NIH/3T3 target cells or measured in a 1:10,000 dilution in an ELISA. In harvests 2 and 3 of VPC MuPACK.e.SB-LEGFP-N1_mRNA_ cells were cultivated in Dynamis™ expression medium. Standard deviations (SD) of mean are indicated.

VPC	Harvest	Viability [%]	Mean titer [TU/mL]	SD	Mean p30 [ng/mL]	SD
MuPACK.e. LEGFP-N1	1	76	9.63 × 10^5^	±0.35 × 10^5^	6.38 × 10^4^	±0.96 × 10^4^
	2	85	1.26 × 10^6^	±0.05 × 10^6^	8.02 × 10^4^	±1.71 × 10^4^
	3	85	2.16 × 10^6^	±0.15 × 10^6^	1.16 × 10^5^	±0.22 × 10^5^
MuPACK.e. SB-LEGFP-N1	1	92	2.17 × 10^6^	±0.11 × 10^6^	3.75 × 10^4^	±1.89 × 10^4^
	2	90	2.66 × 10^6^	±0.21 × 10^6^	5.04 × 10^4^	±0.17 × 10^4^
	3	94	3.21 × 10^6^	±0.32 × 10^6^	4.24 × 10^4^	±1.54 × 10^4^
MuPACK.e. SB-LEGFP-N1_mRNA_	1	80	5.12 × 10^7^	±0.51 × 10^7^	7.20 × 10^4^	±0.78 × 10^4^
	2	73	2.00 × 10^7^	±0.05 × 10^6^	1.86 × 10^5^	±0.24 × 10^5^
	3	90	3.10 × 10^6^	±0.55 × 10^6^	1.12 × 10^5^	±0.01 × 10^5^

**FIGURE 2:**
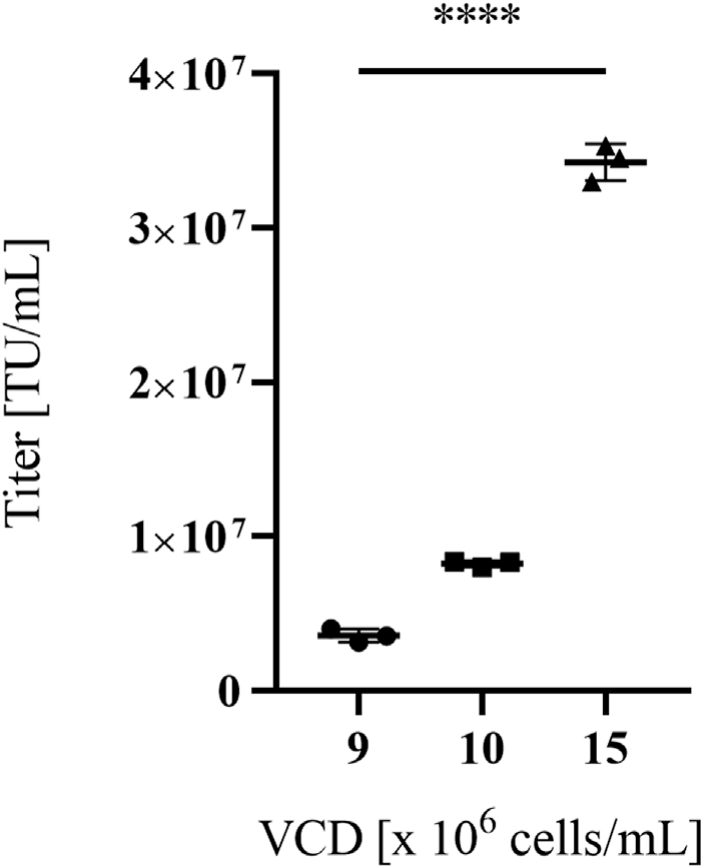
Titers of MLV-based vectors in NIH/3T3 target cells of VPC MuPACK.e.SB-LEGFP-N1_mRNA_. Viral vectors were harvested at three different viable packaging cell densities (VCDs) of 9-, 10- and 15 × 10^6^ cells/mL. Data shown represent values of technical triplicate experiments ± standard deviation. Statistical significance for all three VPCs with n = 3 was determined to *p* ≤ 0.0001(****) using the tailed unpaired Student’s t-test.

When the VPC was expanded in Dynamis™ (harvests 2 and 3), known as one of the mediums of choice for batch- and fed-batch cultivation of highly efficient mammalian producer cells, to prepare for cultivation at high densities in an STR or in perfusion cultivation, cell viabilities varied between 73% and 90% and vector titer productivities of 2.00 × 10^7^ TU/mL and 3.10 × 10^6^ TU/mL were achieved. For high viable cell density cultivations (VCD), represented in Figure 2, VPCs were cultivated at 50 mL scale and vector particle harvests at VCDs of 9, 10 and 15 × 10^6^ cells/mL were titrated in NIH/3T3 cells. VCDs correlated with functional vector titers ranging from 3.58 × 10^6^ TU/mL at 9 × 10^6^ cells/mL to 3.43 × 10^7^ TU/mL at 15 × 10^6^ cells/mL in NIH/3T3 cells.

**FIGURE 3:**
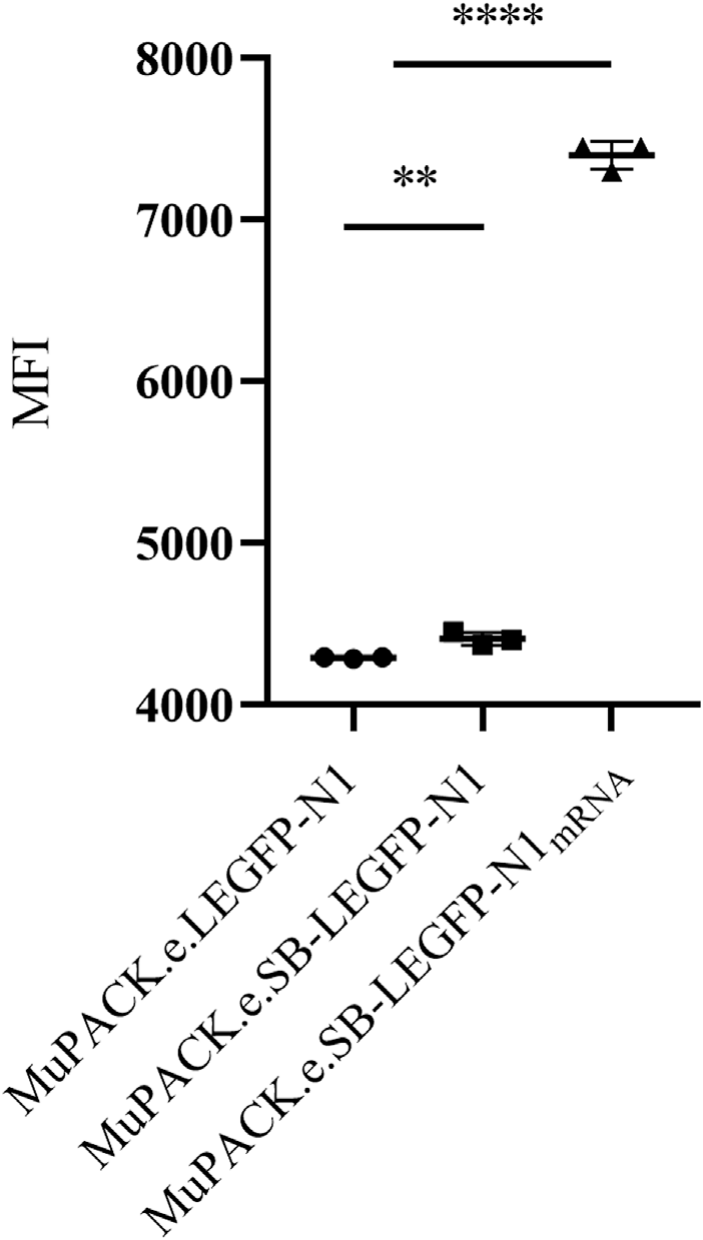
Median fluorescent intensities (MFI) of the three VPCs: MuPACK.e.LEGFP-N1, MuPACK.e.SB-LEGFP-N1 and MuPACK.e.SB-LEGFP-N1_mRNA_. Data shown represent measurements in technical triplicates ±standard deviation. Statistical significance between MuPACK.e.SB-LEGFP-N1 and MuPACK.e.SB-LEGFP-N1_mRNA_ based data with n = 3 was determined to *p* ≤ 0.0001 (****); between MuPACK.e.LEGFP-N1 and MuPACK.e.SB-LEGFP-N1 was *p* ≤ 0.01 (**) using a tailed unpaired Student’s t-test.

These results were supported by the median fluorescence intensities (MFIs) of the three VPCs detected by flow cytometry and represented in Figure 3. The highest expression of EGFP was detected in MuPACK.e.SB-LEGFP-N1_mRNA_ showing a significantly higher MFI of more than 7,000 compared to the two other VPCs with MFIs between 4,000 and 5,000 (*p* ≤ 0.0001).

### Physical titer assessment using p30 capsid-specific ELISA

To evaluate the total amount of MLV capsid protein p30 within the three cell-free VPC harvests, a colorimetric ELISA assay was performed, depicted in Table 3. Average p30 concentrations of all three harvests (not shown in Table 3) were for MuPACK.e.LEGFP-N1 8.65 × 10^4^ ng/mL (±2.65 × 10^4^), for MuPACK.e.SB-LEGFP-N1 4.34 × 10^4^ ng/mL (±0.65 × 10^4^) and for MuPACK.e.SB-LEGFP-N1_mRNA_ 1.23 × 10^5^ ng/mL (±0.58 × 10^5^).

### Detection of transfer vector transcripts in vector particles using RT-qPCR

**FIGURE 4:**
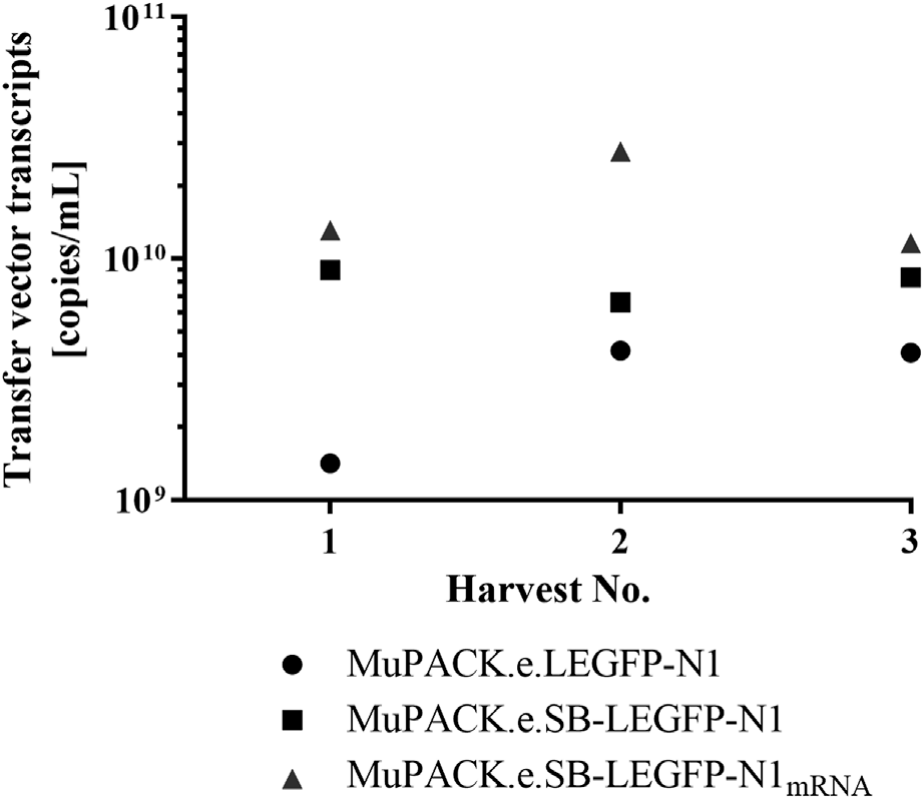
Physical titers of transfer vector transcripts in vector particles measured by real-time RT-qPCR. EGFP-specific primers were used to generate amplicons using LEGFP-N1 mRNA packaged in vector particles in each three harvests of the three VPCs (depicted in Table 3). Mean values from assays performed in analytical duplicates are indicated. Dots: MuPACK.e.LEGFP-N1, rectangles: MuPACK.e.SB-LEGFP-N1, triangles: MuPACK.e.SB-LEGFP-N1_mRNA_.

The different functional vector titers were likely to result from different transfer vector transcript amounts available for packaging into the vector particles. Thus, RT-qPCR was performed in duplicate using frozen cell-free samples from all VPC vector harvests and EGFP-specific primers. As illustrated in Figure 4, the mean amount of mRNA detected confirmed the trend observed in functional vector titers obtained from all three VPCs. MuPACK.e.SB-LEGFP-N1_mRNA_ (1.16 × 10^10^ to 2.78 × 10^10^ copies/mL) revealed the highest amount of packaged transcripts as compared to MuPACK.e.SB-LEGFP-N1 (6.60 × 10^9^ to 8.96 × 10^9^ copies/mL), while MuPACK.e.LEGFP-N1 yielded the lowest amounts of encapsidated mRNA (1.42 × 10^9^ to 4.16 × 10^9^ copies/mL).

### Production and functional titer of MLV vectors in STR

**FIGURE 5:**
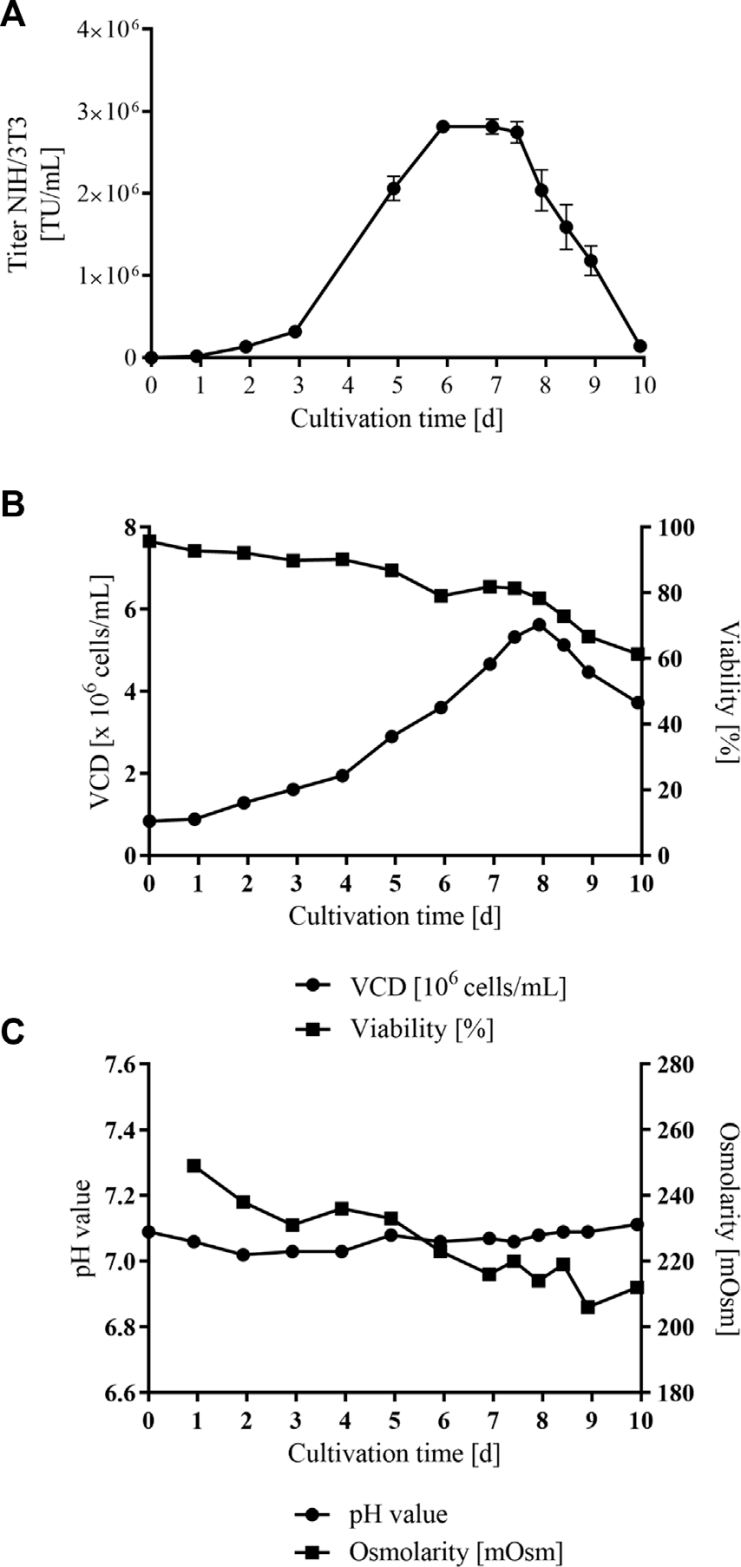
Stirred-tank bioreactor vector particle production over 10 days with VPC MuPACK.e.SB-LEGFP-N1_mRNA_. **(A)** NIH/3T3 cells were transduced with cell-free MLV vector containing supernatants harvested at twelve time points. Data points represent average values of technical triplicates, standard deviations are shown as vertical error bars. **(B)** Viable VPC density (VCD) in 1 × 10^6^ cells/mL (dots) and cell viability in % (rectangles) during a 10-day STR cultivation. **(C)** pH values (dots) and Osmolarity in mOsm (rectangles) during a 10-day STR cultivation.

To enable vector production at a larger scale, a cultivation in STR was examined with the most productive stable VPC MuPACK.SB-LEGFP.N1_mRNA_. Cells were cultivated for 10 days in 500 mL Dynamis™ medium supplemented with 8 mM L-glutamine in the absence of antibiotics and medium change as described in detail in materials and methods as well as in Table 1. Figure 5 shows the retroviral vector titers detected in NIH/3T3 cells during a 10 days STR procedure. The VPC culture revealed increasing productivity up to day 6. As illustrated in Figure 5A and at the onset of the culture process, the titers were rather low with about 1 × 10^5^ TU/mL correlating with the low VCD of less than 2 × 10^6^ cells/mL. With increasing VCDs up to 6 × 10^6^ cells/mL (Figure 5B) the transduction-competent particle numbers also increased, generating titers of up to 2.81 × 10^6^ TU/mL in NIH/3T3 cells at day 6. From day 6 to day 7, productivity stayed on a small plateau and from day 7 on, the VCDs continuously decreased to 3.72 × 10^6^ cells/mL resulting in declining titers of 2.04 × 10^6^ TU/mL down to 1.4 × 10^5^ TU/mL at the end of the STR process on day 10. A linear scalability was observed until day 6 of production remaining then on a plateau. From day 8 on and as no medium exchange was performed, the vector production rate, as well as vector titer, dropped in correlation with the decline in VCDs and viability, respectively. While osmolality slightly decreased over time from 249 to 212 mOsm, pH values remained considerably stable around 7.0 (+deadband) throughout the process (Figure 5C).

## Discussion

To facilitate high vector yield production in larger scale, stable suspension VPCs are indispensable. In the first step and within only 3 weeks, we established the polyclonal ecotropic MLV-derived suspension VPC MuPACK.e using transposon vector components as previously described and shown in efficient transduction experiments in murine myeloblast-like cells as well as in hematopoietic stem and progenitor cells (Berg et al., 2019; van Heuvel et al., 2021). The establishment of stable high-titer producing VPCs co-expressing the transfer vector of choice is a tedious and time-consuming process. To reduce development times, we compared in a proof-of-concept study three stable gene transfer techniques. In one approach i) the transfer vector plasmid pLEGFP-N1 was simply stably transfected. ii) The transfer vector cassette was flanked by SB-derived TIRs and co-transfected with a transposase plasmid construct pSB100X aiming at the stable transposition of the viral vector component into the VPCs genomes. iii) To exclude the undesired stable transfection of pSB100X and the expression of the transposase over a period of one week, pSB-LEGFP-N1 was co-transfected with mRNA-SB100X.

MuPACK.e.LEGFP-N1 produced vectors at high titers of 9.63 × 10^5^ to 2.16 × 10^6^ TU/mL in NIH/3T3 target cells. These results exceed previously reported ecotropic MLV vector titers. Chan et al. established a human lymphoblast WIL-2 cells-derived suspension VPC using conventional plasmid transfection obtaining 7.5 × 10^5^ TU/mL in NIH/3T3 cells (Chan et al., 2001). MuPACK.e.SB-LEGFP-N1 generated only moderately improved titers between 2.17 × 10^6^ and 3.21 × 10^6^ TU/mL.

MuPACK.e.SB-LEGFP-N1_mRNA_ showed drastically increased productivities reaching vector titers of up to 5.12 × 10^7^ TU/mL when cultivated in 100 mL shake flask volumes at a cell density of 4 × 10^6^ cells/mL in FreeStyle™ or in Dynamis™ medium, respectively. In addition, these results were supported by the physical titers detected using an anti-p30 ELISA. MuPACK.e.SB-LEGFP-N1_mRNA_ showed p30 amounts a power of ten higher than the other two VPCs. Contaminations with RCRs resulting from recombination events of the retroviral vector components were not detected in any of the vector particle preparations conducting a GFP-marker rescue assay and a sensitive RT-detection assay (data not shown).

The high abundance of the transfer vector RNA available for packaging in concert with high level expression of Gag/Pol is a crucial prerequisite for the efficient formation of transduction-competent vector particles (Berg et al., 2019; Sweeney and Vink, 2021). Flow cytometric analysis of the three VPCs revealed different expression levels of EGFP, and thus indicating differences in transfer vector transcript amounts. MuPACK.e.SB-LEGFP-N1_mRNA_ showed a significantly higher MFI compared to the two other VPCs.

Consequently and to assess whether these differences also mirrored the copy number of encapsidated transfer vector mRNA, vector particle harvests from all three VPCs were examined using RT-qPCR. MuPACK.e.SB-LEGFP-N1_mRNA_ showed the highest amount of packaged transfer vector RNA followed by MuPACK.e.SB-LEGFP-N1 and MuPACK.e. LEGFP-N1 confirming the trend observed in functional vector titers. However, the RNA levels were two to three orders of magnitude higher than the functional titers in respective target cells. This gap using two different measurements was previously reported by Geraerts and colleagues 2006 (Geraerts et al., 2006). Transfer vector RNAs detected in the supernatant of the VPCs using qPCR are not necessarily encapsidated (Onafuwa-Nuga et al., 2005; Rulli et al., 2007; Eckwahl et al., 2016).

FreeStyle™ medium limits the VCDs to 4 × 10^6^ cells/mL. We thus conducted a high VPC density experiment in Dynamis™ medium in a 50 mL shake flask scale. VCDs could be elevated to 1.5 × 10^7^ cells/mL reaching titers of up to 3.43 × 10^7^ TU/mL in NIH/3T3 cells. This encouraged us to conduct a first STR pilot cultivation. VPC MuPACK.e.SB-LEGFP-N1_mRNA_ was expanded at larger scale using 500 mL volume STR employing Dynamis™. When the VPC was seeded at a VCD of 0.84 × 10^6^ cells/mL, the maximal cell density peaked at 5.62 × 10^6^ cells/mL along with increasing viability. The highest vector titers of up to 2.81 × 10^6^ TU/mL were obtained on day 6. Osmolality and pH values were moderately decreasing and remained stable, respectively, over the entire cultivation period of 10 days. Viral vector titer and viability decline from day 7 on correlated with the decreasing availability of essential nutrients within the expression medium and with an increase of metabolic degradation products. In addition and observed previously, the amount of cellular proteases may have increased, and thus degraded MLV vector particles (Genzel et al., 2010; Petiot et al., 2011; Hein et al., 2021). Therefore, and to reach productivities of >1 × 10^7^ TU/mL in fed-batch approaches, the seed VCD of VPCs should be increased to 2 or 4 × 10^6^ cells/mL allowing the cells to linearly grow to densities of 1.5 × 10^7^ cells/mL or even higher values within the first two or 3 days. Alternatively, fully automated high-density perfusion reactors could be employed, and process parameters would need to be optimized.

Serving as a proof-of-concept, only one VPC pool per transfection technique of the transfer vector was examined here. However, our findings still strongly indicate that the use of transposon-encoding mRNA is superior to the employment of plasmid-based transposase. Using a ratio of 1:1 (transposase transcript to transfer vector plasmid) instead of a ratio of 1:10 (plasmid-based transposase to transfer vector plasmid) for stable transposition presumably led to a higher availability of active transposases, elevating transposition efficiency. The high amount of transposon-encoding mRNA probably resulted in enhanced copy numbers of SB-LEGFP-N1 per cell genome. Transposase transcripts limit transposase expression to about 18 h Bire et al., 2013). Within this time, a high abundance of transiently co-transfected transposon donor plasmids are available and are likely to facilitate the superior transposition. Using mRNA-based transposase expression appears to avoid overexpression inhibition (OPI) or cytotoxicity observed when plasmid-based transposase constructs are used (Grabundzija et al., 2010; Galla et al., 2011; Bouuaert et al., 2013). OPI is most likely a result of high transposase activity over a period of up to 14 days. A prolonged expression of the transposase could lead to re-mobilization and possibly depletion of packaging and envelope donor expression cassettes resulting in less efficient production of viral vector particles.

To date, larger scale retroviral vector productions are mainly done in cell-factories, packed-bed bioreactors or fixed-bed-bioreactors using adherent VPCs. The cells thus grow on a limited area of the stacked cultivation devices or scaffolds such as beads and microfibers (Merten et al., 2001; Wang et al., 2015; Powers et al., 2020). MLV vectors pseudotyped with the Env proteins of Gibbon ape Leukemia virus (GaLV) with titers ranging from 7.88 × 10^5^ up to 3 × 10^7^ TU/mL could be generated using fixed-bed bioreactors in volumes of 200 mL to 1.4 L, respectively (Merten et al., 2001). Using STRs and perfusion reactors instead, generated MLV vectors pseudotyped with GaLV Env and vesicular stomatitis virus G protein (VSV-G) with titers between 5 × 10^5^ and 3.1 × 10^7^ TU/mL, respectively (Merten et al., 2001; Ghani et al., 2006). The packaging cell line PG13 stably transfected with a transfer vector reached titer of 2 × 10^6^ TU/mL and a *piggy-bac* transposon SIN packaging cell line produced titers of up to 3 × 10^6^ TU/mL. A non-transposon-based amphotropic suspension packaging cell line called 293 GP-A2 produced titers of 4 × 10^7^ TU/mL at a VCD of 12 × 10^6^ cells/mL. However the mean time to develop this stable VPC took several months (reviewed in Park et al., 2018).

Ecotropic MLV vectors, as shown here in our proof-of-concept study, were not yet produced at such titers. We thus anticipate that the VPC MuPACK.e and our approach to rapidly establish VPCs within only 3 weeks using mRNA-based transposase transcripts will foster future viral vector productions at larger scale to facilitate preclinical *ex vivo* gene transfer studies into murine primary cells, respectively. The methodology reported here should also be applicable to SIN-transfer vectors harboring a much lower risk for proto-oncogene insertion sites (Hacein-Bey-Abina et al., 2014; Morgan et al., 2021). Transposon vector utilizing strategies should also prove useful to establish VPCs producing vectors with a broadened host cell range utilizing heterologous envelope proteins stemming from the amphotropic molecular clone MLV 4070Amc or dual tropic 10A1mc (Ghani et al., 2007), GaLV, feline endogenous retrovirus RD114 or VSV-G (Ghani et al., 2006; 2009).

## Data Availability

The raw data supporting the conclusion of this article will be made available by the authors, without undue reservation.
